# Glaucocalyxin A enhances colonic anastomotic healing after ischemia–reperfusion: experimental findings

**DOI:** 10.1590/acb412526

**Published:** 2026-05-11

**Authors:** Mehmet Algül, Bariş Rafet Karakaş, Oğuzhan Fatih Ay, Sadika Halide Akbaş, Arzu Karaveli, Kadir Balaban

**Affiliations:** 1 Eskisehir Osmangazi University – Medicine Faculty – Surgical Oncology Department – Eskişehir – Turkey.; 2 Antalya Research and Training Hospital – General Surgery Department – Antalya – Turkey.; 3 Eskisehir Osmangazi University – Medicine Faculty – Gastrointestinal Surgery Department – Eskişehir – Turkey.; 4 Akdeniz University – School of Medicine – Department of Clinical Chemistry – Antalya – Turkey.; 5 Antalya Research and Training Hospital – Anaesthesiology and Reanimation – Antalya – Turkey.; 6 Antalya Training and Research Hospital – Clinic of Pathology – Antalya – Turkey.

**Keywords:** Reperfusion Injury, Colorectal Surgery, Oxidative Stress, Rats

## Abstract

**Purpose:**

Glaucocalyxin A (GLA), a diterpenoid with antioxidative and anti-inflammatory properties, has shown vascular protective effects in preclinical models. This study aimed to evaluate whether GLA improves colonic anastomotic integrity following ischemia–reperfusion (I/R) injury.

**Methods:**

Twenty-four female Sprague Dawley rats were randomized into three groups (n = 8): control + GLA, I/R, and I/R + GLA. I/R was induced by 60-minute superior mesenteric artery occlusion and 60-minute reperfusion before end-to-end colonic anastomosis. GLA (10 mg/kg) was administered intraperitoneally. On postoperative day 7, anastomotic bursting pressure, adhesion scores, hydroxyproline, oxidative stress markers (total oxidative status, total antioxidant capacity, ischemia-modified albumin), and histopathology were evaluated.

**Results:**

The I/R + GLA group demonstrated significantly higher bursting pressure compared with the I/R group (126 versus 40 mmHg; *p* < 0.005). Tissue TOS levels were significantly lower in the I/R + GLA group compared with both other groups (*p* < 0.005). No significant differences were found in hydroxyproline levels, IMA values, or histopathological healing scores.

**Conclusion:**

GLA may support colonic anastomotic healing after I/R injury by reducing oxidative stress and could serve as a potential protective agent against anastomotic compromise.

## Introduction

Colorectal anastomosis is a cornerstone of gastrointestinal surgery, frequently performed in both benign and malignant diseases. Anastomotic leakage (AL) remains one of the most critical and potentially life-threatening complications following colorectal surgery, with incidence rates ranging from 5 to 19% depending on patient comorbidities, anastomotic location, and technical variables^1^.

AL is associated with increased morbidity, prolonged hospitalization, delayed adjuvant treatment in oncologic cases, and significantly higher mortality rates. From an economic perspective, the occurrence of AL imposes a substantial burden on healthcare systems. The mean additional length of hospital stay due to AL has been reported as approximately 10 days, with a range extending up to 20 days in some European studies. In the United Kingdom, the average cost per AL case is estimated at £ 17,000, translating into an annual national healthcare expenditure of £ 1–3 million^2^. Similarly, in the United States of America, AL incurs an additional $ 24,000 in costs per patient and prolongs hospitalization by approximately seven days^3^.

A wide spectrum of risk factors has been associated with colorectal AL, broadly categorized into patient-related and surgery-related determinants^4^. Patient-specific factors include male sex^5^, age over 60, ASA scores ≥ III^6^, malnutrition (particularly hypoalbuminemia)^7^, anemia, obesity, smoking, renal and cardiovascular disease, immunosuppression, and the use of neoadjuvant chemoradiotherapy^4^. These factors are often linked to impaired collagen synthesis, poor immune response, or compromised tissue oxygenation—all critical components of intestinal healing. Among surgical and perioperative contributors, emergency procedures, multiple stapler firings7, prolonged operative time, intraoperative bleeding^8^, and preoperative transfusions have been recurrently reported. Additionally, the anatomical site of the anastomosis, particularly low rectal anastomoses, and technical aspects such as tension, contamination, or repeated stapling are also considered high-risk^9^.

It has been demonstrated that adequate tissue perfusion and oxygen transport are among the most fundamental determinants of anastomotic healing, particularly at colonic suture lines^10^. Ischemia is defined as a state in which insufficient blood flow fails to meet the metabolic requirements of cells, tissues, or organs, potentially leading to reversible or irreversible injury depending on the duration and severity of oxygen deprivation^11,12^. Restoration of blood flow—termed reperfusion—may paradoxically result in additional damage. This is primarily due to the abrupt reintroduction of molecular oxygen, which generates reactive oxygen species (ROS) within the ischemic tissue^13^. These oxygen-derived free radicals are directly responsible for lipid peroxidation, membrane disruption, and cellular apoptosis, thereby constituting the core pathophysiological mechanism of ischemia-reperfusion (I/R) injury.

Clinically, I/R injury is frequently encountered in surgical practice, particularly in settings such as mesenteric vascular occlusion, trauma surgery, volvulus, strangulated hernias, and inflammatory bowel diseases including ulcerative colitis and Crohn’s disease^14^. In the context of colonic anastomosis, I/R-induced oxidative and inflammatory damage impairs collagen synthesis and submucosal matrix integrity, thereby predisposing to anastomotic dehiscence, prolonged hospitalization, and potentially fatal outcomes^15^.

Glycocalyxin A (GLA) is a synthetic polysaccharide compound designed to preserve the structural integrity of the endothelial glycocalyx, a critical regulator of vascular permeability and inflammation. Its protective effect has been demonstrated in I/R injury models, in which it reduces endothelial damage by inhibiting glycocalyx degradation, improving microcirculatory stability, and modulating oxidative stress responses^16^.

The objective of this study was to evaluate the protective effects of GLA on the mechanical, biochemical, and histopathological healing parameters of colon anastomosis in a rat model of I/R. By targeting the glycocalyx and oxidative pathways, we aimed to determine whether GLA could serve as a novel therapeutic strategy for enhancing anastomotic integrity in compromised tissue environments.

## Methods

### Animals and experimental design

This prospective, randomized, and controlled *in-vivo* experimental study was conducted at the Akdeniz University Faculty of Medicine, Laboratory of Experimental Animals, in accordance with the Animal Research: Reporting of In Vivo Experiments (ARRIVE) guidelines. The study protocol was reviewed and approved by the Akdeniz University Local Ethics Committee for Animal Experiments (Approval No.: B.30.2.AKD.05.07.00/9, on January 24, 2018).

Initially, a total of 32 female Sprague Dawley rats (aged 8–10 weeks old, weighing 180–220 g) were enrolled. Animals were housed in temperature- and humidity-controlled rooms (22 ± 2 °C, 60% humidity) under a 12-hour light/dark cycle with *ad libitum* access to standard chow and water. Based on the predefined exclusion criteria, eight animals were excluded from the final analysis: three rats due to intraoperative anesthetic complications and technical failures, and five rats due to postoperative mortality before reaching the seven-day endpoint.

- Group 1 (sham + GLA): mesenteric vessel isolation without occlusion followed by colonic anastomosis and 10 mg/kg GLA;
- Group 2 (I/R + DMSO): 60 min ischemia and 60 min reperfusion followed by colonic anastomosis and 0.2 mL/kg DMSO;
- Group 3 (I/R + GLA): 60 min ischemia and 60 min reperfusion followed by colonic anastomosis and 10 mg/kg GLA;

### Surgical procedure and ischemia-reperfusion model

All surgical procedures were performed under general anesthesia induced by intraperitoneal injection of 100 mg/kg ketamine hydrochloride (Ketalar; Eczacıbaşı, Istanbul, Turkey) and 10 mg/kg xylazine (Rompun; Bayer, Istanbul, Turkey). Following a 3–4-cm midline laparotomy and povidone-iodine antisepsis, the bowel loops were exteriorized and kept moist with saline-soaked sterile gauze.

In the I/R groups, mesenteric vessels of a 2–3 cm segment of the left colon were isolated and ligated with 2/0 vicryl for 60 minutes. Ischemia was clinically confirmed by the observation of paleness in the colonic wall and the loss of arterial pulsation. Following the 60-minute ischemic period, the ligatures were removed for 60 minutes of reperfusion, which was verified by the bowel regaining a healthy, pinkish color. After the I/R cycle, colonic transection was performed, followed by end-to-end anastomosis using 5/0 polypropylene sutures. All animals received 10 mL of intraperitoneal saline during surgery to prevent dehydration.

The rats in the control GLA and I/R GLA groups received GLA (10 mg/kg) via intraperitoneal (i.p.) injection. The dosage was determined based on previous studies in the literature^17^ demonstrating its effective antioxidant and anti-inflammatory properties in intestinal models.

### Anastomotic bursting pressure measurement

On postoperative day 7, all rats underwent re-laparotomy under the same anesthesia protocol. Peritoneal adhesions were scored according to the Evans classification (0–3). A 4-cm colonic segment including the anastomosis was resected and cleared of fecal matter.

The proximal end was connected to an infusion pump (Perfusor Compact; B. Braun, Melsungen, Germany), and the distal end was linked to a pressure transducer and monitor (Datex–Ohmeda S/5; GE Healthcare, United Kingdom). Methylene blue-stained saline was infused at a constant rate of 2 mL/min until a leak occurred at the anastomotic line. The peak pressure at the moment of the leakage was recorded as the bursting pressure (mmHg). Following the completion of sample collection, all rats were euthanized via cardiac exsanguination under deep anesthesia.

### Biochemical analysis

Tissue and serum samples were analyzed at the Akdeniz University Department of Biochemistry. Total oxidative status (TOS) and total antioxidant capacity (TAC) levels were measured using automated colorimetric methods developed by Erel. Tissue ischemia-modified albumin (IMA) and hydroxyproline levels were determined using YLBIONT rat enzyme linked immunosorbent assay (ELISA) kits (Cat No: YLA0290RA and YLA0068RA, respectively) via the sandwich ELISA method. All tissue results were normalized to the total protein concentration, determined via the Bradford method, and expressed as units per gram of protein (U/g protein) or amount per gram of protein.

### Pathological evaluation

Anastomotic segments fixed in 10% buffered formalin were embedded in paraffin and stained with hematoxylin-eosin and Masson’s trichrome. A blinded pathologist evaluated the specimens using the modified Ehrlich and Hunt scoring system (0–4) to grade inflammatory cell infiltration, fibroblastic activity, neovascularization, and collagen deposition.

### Sample size

Each group included eight rats, with a total of 24 rats used in the entire experiment.

A formal *a priori* power calculation was not performed. The group size was based on similar previous studies reported in the literature to be sufficient to observe statistically significant differences in bursting pressure and biochemical markers.

### Inclusion and exclusion criteria

Inclusion criteria were:

- Female Sprague Dawley rats aged 8–10 weeks old, weighing between 180–220 grams;
- Animals deemed healthy upon preoperative clinical evaluation;
- Animals that tolerated anesthesia and surgical procedures without intraoperative complications (e.g., excessive bleeding, bowel perforation not related to experimental design);
- Animals that completed the entire postoperative seven-day follow-up period without signs of sepsis or major systemic deterioration.
  Exclusion criteria were:
- Preoperative illness or failure to meet the weight range criteria;
- Intraoperative mortality or technical complications (n = 3): included anesthetic overdose and iatrogenic bowel injury during mesenteric vessel isolation;
- Postoperative mortality or severe complications (n = 5): included bowel obstruction, wound dehiscence, or mortality before reaching the seven-day endpoint;
- Animals with incomplete data collection due to procedural failures.

### Randomization

Rats were randomly allocated to each group (1:1:1) using simple randomization.

No additional measures were taken to control for environmental confounders such as cage location or time of surgery; all procedures were conducted under standardized environmental conditions.

### Blinding

Outcome assessment, including measurement of bursting pressure and histopathological scoring, was performed by investigators who were blinded to group allocation. However, due to the nature of the interventions, it was not possible to blind the surgical operator.

### Outcome measures and statistical analysis

The primary outcome of the study was the anastomotic bursting pressure measured on postoperative day 7, which served as the principal mechanical indicator of anastomotic healing. In addition, several secondary outcome measures were evaluated to assess both biochemical and histopathological aspects of healing. These included tissue hydroxyproline concentration as a surrogate marker for collagen synthesis; TOS and TAC measured in both tissue and serum samples to assess oxidative balance; and IMA levels in tissue homogenates to reflect ischemia-induced biochemical changes. Furthermore, histopathological healing was scored based on inflammatory cell infiltration, fibroblastic activity, neovascularization, and collagen deposition, using the modified Ehrlich and Hunt scoring system under light microscopy.

All statistical analyses were conducted using Statistical Package for the Social Sciences version 19.0 (Chicago, IL, United States of America). Data were summarized as median values with minimum and maximum ranges. Comparisons between groups were performed using the Kruskal–Wallis’ test, while Mann–Whitney’s U-test was applied for post hoc pairwise analyses. A *p* ≤ 0.005 was considered statistically significant. Given the use of non-parametric statistical methods, no formal assessment of normality was conducted.

## Results

Table 1 summarizes the comparative findings of adhesion scores, bursting pressure, serum, and tissue oxidative stress parameters, hydroxyproline and IMA levels, and histopathological grading among the study groups. A statistically significant difference was observed in anastomotic bursting pressure and tissue total oxidant status (T-TOS) between the I/R group and both the control GLA and I/R GLA groups (*p* = 0.001 and *p* = 0.002, respectively), suggesting the potential protective role of GLA in maintaining anastomotic integrity under I/R conditions.

**Table 1:** Evaluation of adhesion scores, bursting pressure measurements, biochemical analysis results, and histopathological grading across the experimental groups^<tfn href="tfn01">#</tfn>^.

	Sham + GLA group	Ischemia/reperfusion group	Ischemia/reperfusion + GLA group
**Adhesion score**	2 (1–3)	2 (1–3)	1,5 (1–3)
**Bursting pressure (mmHg)**	67 (47–151)*	40 (26–57)* ^¶^	**126 (45–167)** ^¶^
**Biochemical analysis**			
S-TOS (µmol/L)	19.1 (15.3–23.1)	17 (14–45.4)	26.1 (15.3–191)
S-TAC (µmol/L)	1.8 (1.5–2.1)	1.8 (0–2.3)	1.2 (0–2.1)
Tissue hydroxyproline level	14.1 (7.8–26.6)	22 (10.5–38.5)	14 (10.1–21.1)
Hydroxyproline/protein ratio (µg/mg protein)	1,801.1	2,950.6	1,611.6
IMA (U/g protein)	(1,037–4,016)	(1,159.6–5,811.5)	(1,129–2,491.2)
T-TOS (µmol/g protein)	**1.4 (1.1–2.2)** ^∆^	**1.3 (1–1.9)** ^¶^	**1 (0.8-1.9)** ^¶ ∆^
T-TAC (µmol/g protein)	0 (0–0.1)	0.1 (0–0.3)	0 (0–0.1)
**Histopathological evaluation**			
Inflammatory cell infiltration	2.5 (2–4)	3 (2–4)	2 (1–4)
Neovascularization	2 (1–3)	2.5 (2–4)	2 (1–3)
Fibroblast activity	2 (1–3)	3 (1–4)	2 (1–4)
Collagen deposition	2 (2–3)	2.5 (2–4)	2 (1–3)

# Adhesion scores, anastomotic bursting pressure, biochemical markers, and histopathological healing parameters are presented as median values with ranges (minimum–maximum). Statistical analysis was performed using the Kruskal–Wallis’ test followed by the Mann–Whitney’s U test for pairwise comparisons. A *p* < 0.005 was considered statistically significant; GLA: Glaucocalyxin A;

* *p* < 0.005: comparison between the sham + GLA group and the ischemia/reperfusion group;

¶ *p* < 0.005: comparison between the ischemia/reperfusion group and the ischemia/reperfusion + GLA group;

∆ *p* < 0.005: comparison between the sham + GLA group and the ischemia/reperfusion + GLA group; S-TOS: serum total oxidant status (μmol/L); S-TAC: serum total antioxidant capacity (μmol/L); T-TOS: tissue total oxidant status (μmol/g protein); T-TAC: tissue total antioxidant capacity (μmol/g protein); IMA: ischemia-modified albumin (U/g protein); hydroxyproline/protein ratio, tissue collagen content (µg/mg protein).

Source: Elaborated by the authors.

**Figure 1:**
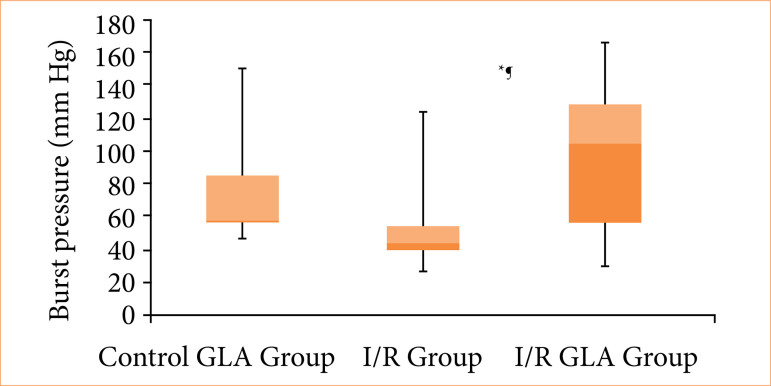
Graphical representation of anastomotic bursting pressure values across all experimental groups#.

When anastomotic bursting pressure values were compared, the sham + GLA group showed significantly higher pressures than the I/R group (*p* < 0.005). Similarly, the I/R + GLA group demonstrated significantly higher bursting pressures compared to the I/R group (*p* < 0.005). However, no statistically significant difference was observed between the sham + GLA and I/R + GLA groups (Fig. 1).

#*p* < 0.005 was considered statistically significant. Statistical analysis was performed using the Kruskal–Wallis’ test for overall group comparisons and the Mann–Whitney’s U test for pairwise comparisons; GLA: Glaucocalyxin A; *p < 0.005 for sham + GLA versus ischemia-reperfusion; ^¶^ *p* < 0.005 for ischemia-reperfusion versus I/R + GLA. Source: Elaborated by the authors.

**Figure 2:**
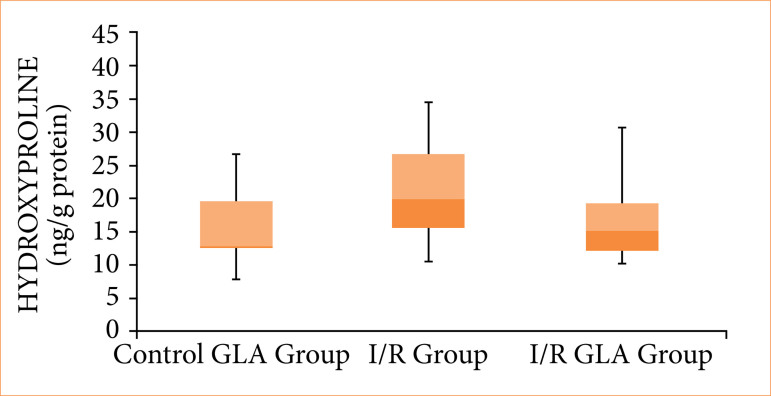
Graphical representation of tissue hydroxyproline levels across all experimental groups.

No statistically significant difference in tissue hydroxyproline levels was observed among the sham + GLA, I/R, and I/R + GLA groups (Fig. 2).

#*p* < 0.005 was considered statistically significant. Tissue hydroxyproline levels were compared using the Kruskal–Wallis test for overall group comparisons; GLA: Glaucocalyxin A; I/R: ischemia-reperfusion. Source: Elaborated by the authors.

**Figure 3:**
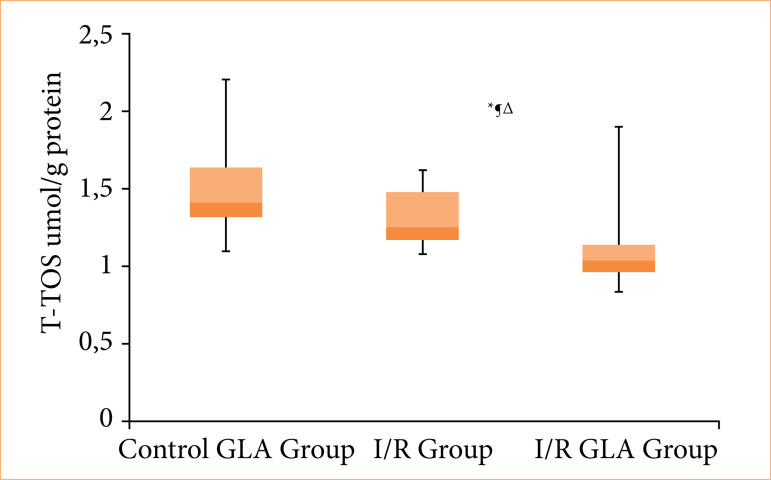
Graphical representation of tissue total oxidant status (T-TOS) values across all experimental groups*.

When comparing tissue TOS levels, the I/R + GLA group showed significantly lower values compared to both the I/R group and the sham + GLA group (*p* < 0.005 for both comparisons). No statistically significant difference was observed between the sham + GLA group and the I/R group (Fig. 3).

*p < 0.005 was considered statistically significant. Statistical analysis was performed using the Kruskal–Wallis’s test for overall comparisons and the Mann–Whitney’s U test for pairwise comparisons; GLA: Glaucocalyxin A; ^¶^ *p* < 0.005 for ischemia-reperfusion versus ischemia-reperfusion + GLA; ^∆^ *p* < 0.005 for sham + GLA versus ischemia-reperfusion + GLA.Source: Elaborated by the authors.

## Discussion

The principal finding of our experimental study is that GLA administration significantly improved anastomotic bursting pressure and reduced TOS in a rat model of I/R-induced colonic injury. Notably, the anastomotic bursting pressure values in the I/R + GLA group were statistically higher compared to the I/R group, indicating a potential protective effect of GLA on anastomotic integrity. Additionally, the significant reduction in tissue TOS levels suggests that GLA may exert its beneficial effects through attenuation of oxidative stress, a well-known contributor to reperfusion-related tissue injury. In contrast, no significant differences were observed in hydroxyproline concentrations, IMA, or histopathological healing scores among the experimental groups. These findings collectively suggest that GLA has the potential to enhance mechanical healing of colonic anastomoses primarily by modulating oxidative balance rather than stimulating collagen deposition or altering histological inflammation in the acute phase of healing.

I/R injury is a critical factor impairing anastomotic healing, especially in colorectal surgery in which segmental resections often require vascular clamping and manipulation. The temporary cessation and subsequent restoration of blood flow results in oxidative stress, neutrophil infiltration, inflammatory cytokine release, endothelial dysfunction, and extracellular matrix degradation, all of which compromise the mechanical integrity of the anastomotic line. This pathophysiological cascade has been investigated in numerous experimental studies using pharmacologic agents to mitigate its effects.

Montelukast, a leukotriene receptor antagonist, was shown to enhance bursting pressure, hydroxyproline levels, and vascularization following I/R injury, indicating its anti-inflammatory benefit on anastomotic healing^18^. Similarly, simvastatin restored both anastomotic strength and collagen content, likely through its antioxidant and endothelium-stabilizing effects when administered postoperatively^19^. Another antioxidant compound, lupeol, reduced pro-inflammatory cytokines, specifically tumor necrosis factor-alpha (TNF-α) and interleukin-6 (IL-6), increased glutathione levels, and improved histological healing, suggesting both biochemical and micro ribonucleic acid (microRNA) protection against I/R insult^20^. Genistein, an isoflavonoid, likewise improved bursting pressures and upregulated superoxide dismutase and glutathione peroxidase, supporting its role in oxidative stress regulation and collagen synthesis^21^.

Ethyl pyruvate, with known ROS-scavenging capacity, significantly increased both bursting pressure and hydroxyproline content when administered post-I/R, regardless of dosage frequency^22^. N-acetylcysteine (NAC), either i.p. or orally, preserved anastomotic strength and enhanced collagen deposition, confirming its protective role against I/R-induced tissue damage^23^. Lastly, studies involving papaverine and pentoxiphylline highlighted the dual role of antioxidant and vasodilatory mechanisms. While pentoxiphylline reduced vascular endothelial growth factor (VEGF) expression and elevated zinc levels, suggesting oxidative control, its anti-angiogenic effect raises concerns regarding its role in mucosal regeneration^24^.

Collectively, these studies illustrate the multifactorial impact of I/R injury on colorectal anastomosis, emphasizing oxidative imbalance, endothelial dysfunction, and impaired collagen remodeling as central mechanisms. Pharmacological interventions targeting these pathways have demonstrated promising results in improving anastomotic integrity. Yet, standardization of I/R models and validation of these agents in translational research remain imperative for clinical application.

When comparing the methodology of our experimental model with prior studies evaluating colorectal anastomotic integrity following I/R injury, it is apparent that while several reports share fundamental mechanical and biochemical assessment techniques, methodological diversity persists in terms of reperfusion duration, treatment timing, and outcome measures. Similar to our design, many studies employed anastomotic bursting pressure^18–20^ and tissue hydroxyproline concentration^22,23^ as primary endpoints to evaluate mechanical strength and collagen deposition.

In terms of histological evaluation, our study applied hematoxylin-eosin staining to assess key tissue repair parameters—specifically inflammatory cell infiltration, neovascularization, fibroblast density, and collagen deposition—through a semi-quantitative scoring system interpreted by a blinded pathologist. This approach is comparable to prior experimental models that also utilized blinded histological grading to assess key parameters such as inflammation, vascular proliferation, and collagen deposition. The Montelukast study, for instance, incorporated both hematoxylin-eosin and Sirius Red staining and scored multiple histological domains including edema, vascular proliferation, and immune cell infiltration on a 0–3 scale^18^. Similarly, the NAC model employed an extended histological protocol using additional stains (Verhoeff-Van Gieson and Masson’s trichrome) and a modified Ehrlich-Hunt scale to evaluate fibroblast activity, vascularity, and collagen deposition^23^. While these studies incorporated broader or more specialized staining techniques, our methodology remains aligned with standard histological assessment strategies used in experimental I/R-anastomosis models.

Additionally, histopathological healing scores were integrated in a subset of studies, which aligns with our comprehensive assessment strategy. However, certain models introduced further dimensions—such as oxidative stress markers (*e.g*., malondialdehyde (MDA), superoxide dismutase (SOD)^21^, glutathione peroxidase, inflammatory cytokines (*e.g.*, TNF-α)^20^, IL-6, or angiogenesis-related factors (e.g., VEGF)^24^—, which were not central in our protocol. Notably, a few studies varied in their reperfusion durations (1–24 hours) and postponed anastomosis^20,21^, while ours applied immediate reanastomosis following a standardized I/R cycle. Thus, our study stands out for its structured integration of both conventional and histological parameters under a tightly controlled I/R setting, while remaining within the bounds of reproducibility and translational relevance.

Numerous preclinical studies have sought to improve our understanding of anastomotic healing and reduce leakage rates in colorectal surgery using experimental models. In a murine colitis model, a standardized and reproducible technique allowed detailed observation of healing under inflammatory conditions, revealing the impact of local disease on anastomotic integrity^25^.

Another study evaluated TISSEEL (fibrin sealant; Baxter Healthcare, Deerfield, IL, United States of America) application in diabetic rats, demonstrating enhanced neovascularization, fibroblast activity, and collagen formation, even under hyperglycemic stress^26^. Complementing these animal data, a human fibroblast-based *in-vitro* model showed that transforming growth factor-beta 1 (TGF-β1) promotes collagen synthesis regardless of colonic or rectal origin, suggesting therapeutic potential in collagen-deficient anastomotic sites^27^. Attempts to deliver cytostatic agents i.p. via hydrogel systems failed to show benefit and raised concerns regarding safety due to hemorrhagic complications^28^. Dietary modulation using inulin and galacto-oligosaccharides improved epithelial regeneration and reduced matrix metalloproteinases activity in mouse models, pointing to the microbiota’s role in mucosal recovery^29^. Human oral mucosa-derived stem cells were shown to reduce leak rates and improve postoperative wellness scores in a colonic anastomosis murine model^30^. Finally, a porcine study analyzing fully covered self-expandable metal stents over stapled anastomoses found transient metabolic alterations without definitive signs of ischemia, raising ongoing questions about mechanical support strategies^31^. These experimental models collectively demonstrate the diversity of strategies evaluated to enhance anastomotic healing in colorectal surgery. Despite this variety, a substantial need for novel agents with regenerative potential and for well-designed preclinical studies to investigate their efficacy under standardized conditions remains.

GLA is an ent-kauranoid diterpene compound (7α,14β-dihydroxy-ent-kaur-16-en-3,15-dione) derived from Rabdosia japonica variety glaucocalyx, a medicinal herb traditionally used across East Asia for the treatment of gastrointestinal diseases and various malignancies. GLA has been reported to possess multiple pharmacological properties, including antibacterial, antioxidative, and anti-neuroinflammatory effects^32,33^. It has also been investigated for its potential therapeutic roles in ischemic diseases, leukemia, neurodegenerative conditions, and breast cancer^16^. Given its broad pharmacological spectrum and traditional applications, our study aimed to evaluate the effects of GLA on colonic anastomotic healing following I/R injury in a controlled rat model.

The potential protective mechanisms of GLA on anastomotic healing in the setting of ischemia-reperfusion injury may involve antioxidative^34^, anti-inflammatory, immunomodulatory^35^, and anti-thrombotic^36^ pathways. GLA has been demonstrated to exhibit antioxidative activity by inhibiting ROS–induced deoxyribonucleic acid (DNA) damage and lipid peroxidation, which are key contributors to I/R–mediated tissue injury. This effect is particularly relevant in colonic anastomoses, in which oxidative stress may impair mucosal integrity and collagen synthesis during the early healing phase. Furthermore, GLA exerts anti-thrombotic and anti-coagulative effects by prolonging thrombin time (TT), prothrombin time (PT), and activated partial thromboplastin time (APTT), alongside attenuating platelet aggregation through modulation of thromboxane A2 (TXA2) and prostaglandin E2 (PGE2) pathways. These actions may support anastomotic perfusion by reducing microvascular thrombosis, a known contributor to I/R-related anastomotic failure. Additionally, GLA has been shown to downregulate pro-inflammatory cytokines (e.g., TNF-α, IL-1β, IL-6) and inhibit nuclear factor kappa-light-chain-enhancer of activated B cells (NF-κB) signaling in microglial and lymphocytic models, suggesting a systemic immunomodulatory and anti-inflammatory profile. In the context of I/R injury, this cytokine modulation could attenuate neutrophil infiltration and inflammatory tissue degradation at the anastomotic site. Collectively, these mechanisms support the hypothesis that GLA may enhance anastomotic healing by mitigating oxidative, inflammatory, and thrombotic cascades typically exacerbated by I/R processes.

GLA has recently garnered attention for its therapeutic potential across various disease models. In inflammatory bowel disease, it has been shown to alleviate colitis by inhibiting the phosphoinositide 3-kinase / protein kinase B / mammalian target of rapamycin (PI3K/AKT/mTOR) signaling pathway^37^. In sepsis, GLA protects liver function by suppressing excessive platelet activation and complement-mediated tissue injury^38^. Moreover, in carbon tetrachloride-induced liver fibrosis, GLA has demonstrated anti-inflammatory and anti-apoptotic effects while also restoring gut microbiota balance^39^. These findings support the anti-inflammatory, antithrombotic, and organ-protective properties of GLA and provide a rational basis for its experimental use in I/R –related tissue injury.

Anastomotic failure remains one of the most critical complications following colorectal surgery, often associated with significant morbidity and mortality. I/R injury has been recognized as a key pathophysiological mechanism impairing anastomotic healing. Considering the ongoing efforts in experimental research to enhance anastomotic integrity, our findings may offer additional insight into the protective role of GLA in this context. While the current literature on GLA is still limited, its multifaceted pharmacological properties—particularly its antioxidative and anti-thrombotic effects—may warrant further exploration in surgical models of intestinal healing. Future studies focusing on dose optimization, delivery methods, and long-term outcomes are essential before clinical application can be envisioned.

This experimental study has several limitations. First, as with all animal models, the physiological differences between rats and humans limit the direct translatability of the results to clinical practice. Second, the sample size, while statistically justified, may not fully reflect the zheterogeneity of biological responses in a broader population. Third, although histopathological and biochemical parameters were comprehensively assessed, molecular-level mechanisms such as gene expression or oxidative stress markers were not evaluated. Additionally, only a single dose and administration route of GLA were investigated, which may not represent the full therapeutic potential or safety profile of the compound. Finally, the short-term follow-up period limited our ability to assess long-term anastomotic outcomes such as stricture formation or delayed leakage.

## Conclusion

This experimental study investigated the effects of GLA on colonic anastomotic healing following I/R injury. Our findings suggest that GLA may exert protective effects through its antioxidative, anti-inflammatory, and anti-thrombotic properties, potentially supporting tissue recovery in compromised anastomotic environments. While these results are encouraging, they should be interpreted within the limitations of an animal model. Further research is needed to validate these findings and explore their clinical relevance in human colorectal surgery.

## Data Availability

The datasets generated and/or analyzed during the current study are available from the corresponding author on reasonable request.
